# Outcome measures in Cochrane reviews and protocols for the prevention and treatment of periodontal disease

**DOI:** 10.1186/1745-6215-16-S1-P32

**Published:** 2015-05-29

**Authors:** Thomas J Lamont, Jan E Clarkson, Craig R Ramsay

**Affiliations:** 1 Dental Health Services Research Unit, Dundee Dental School, the University of Dundee, UK; 2 School of Dentistry, University of Manchester, United Kingdom; 3 Health Services Research Unit, University of Aberdeen, Aberdeen, UK

## Background

Periodontal disease (gum disease) is the most common oral disease to affect adults. The importance of core outcome sets for effectiveness trials is well established. However, despite the significant health and economic burden there is still great debate in the field as to the most appropriate clinical outcomes to measure when investigating the effectiveness & cost effectiveness of interventions. The recently published Scottish Dental Clinical Effectiveness Programme Guidelines “Prevention and Treatment of Periodontal Diseases in Primary Care” [1], highlighted the need for a set of core outcome measures for periodontal outcomes. This study was conducted to assess the variability of outcome measures reported in periodontal Cochrane reviews and protocols prior to the development of a set of core outcomes involving patients and key stakeholders.

## Method

Published Cochrane reviews and protocols (search up to August 2014) investigating the prevention and treatment of periodontal disease were assessed. The type of intervention, outcome measures (clinical, patient and economic) and duration of follow up was recorded. From included trials all indices reported were also noted.

## Results

**Figure 1:**
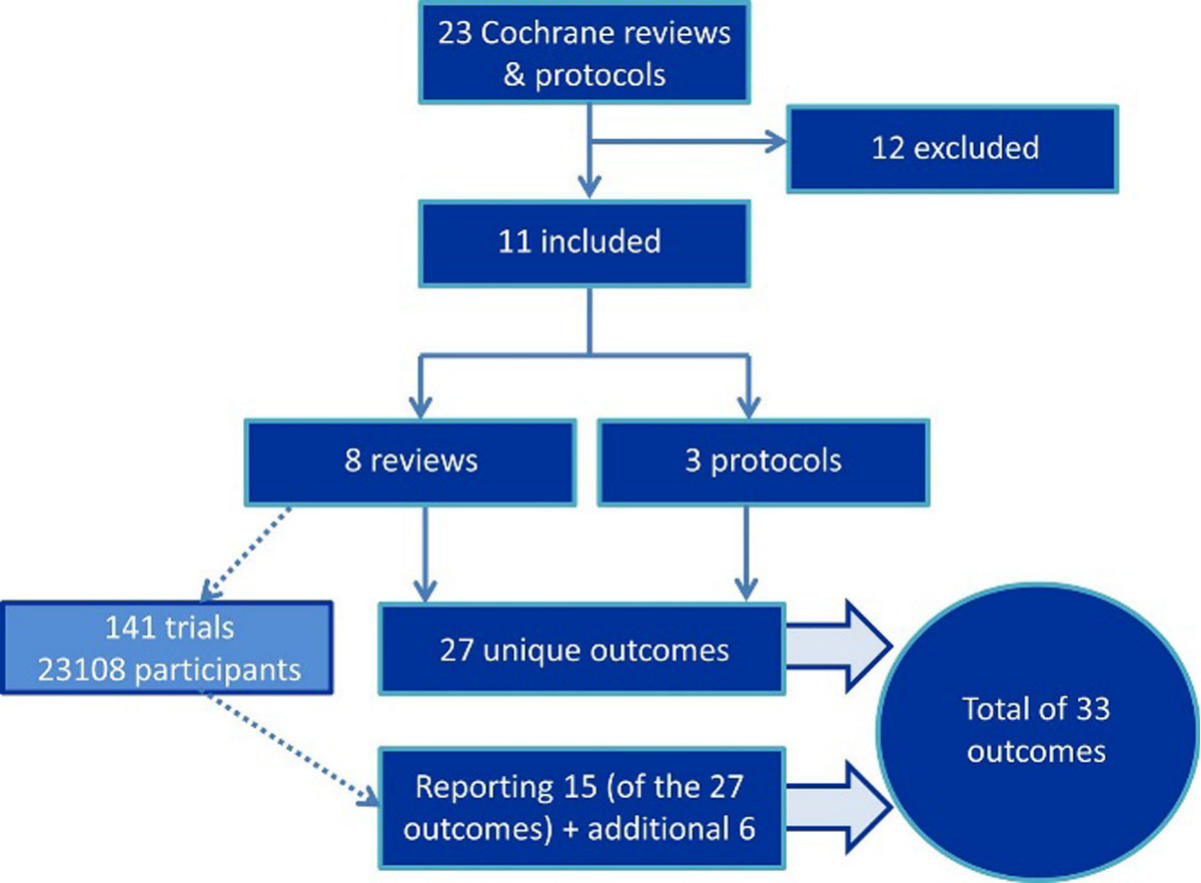
Study flow diagram

We identified 23 systematic reviews and protocols regarding periodontal disease published by the Cochrane Oral Health Group. This study included 8 Cochrane reviews, involving 141 trials with 23,108 patients and 3 protocols (Figure 1). Descriptions of outcomes were varied but, following deduplication 27 unique outcomes were identified, as either primary or secondary, in the Cochrane reviews/protocols. In the published reviews 15 of these 27 outcomes had data available and in the included trials an additional 6 reported outcomes were found. Gingivitis and plaque (surrogate outcomes) were the most commonly reported outcomes and were measured by 17 different indices in 110 and 117 trials respectively. None of the studies reported economic outcome measures. None of the reviews or trials reported to include patients, hygienists, general dental practitioners or policy makers in the outcome measure selection.

## Conclusion

This study demonstrates the variety of outcome measures available for periodontal studies and highlights the number of indices used to record them. There is ongoing debate as to which outcomes should be investigated; reinforcing the need of a core set of outcomes for periodontal studies whilst also highlighting the need for agreed measurement instruments.
